# Model-based correction of rapid thermal confounds in fluorescence neuroimaging of targeted perturbation

**DOI:** 10.1117/1.NPh.11.1.014413

**Published:** 2024-02-16

**Authors:** Neda Davoudi, Hector Estrada, Ali Özbek, Shy Shoham, Daniel Razansky

**Affiliations:** a University of Zurich, Institute for Biomedical Engineering and Institute of Pharmacology and Toxicology, Faculty of Medicine, Zurich, Switzerland; b ETH Zurich, Institute for Biomedical Engineering, Department of Information Technology and Electrical Engineering, Zurich, Switzerland; c NYU Langone Health, Neuroscience Institutes, Department of Ophthalmology and Tech4Health New York, United States; d ETH AI Center, Zurich, Switzerland

**Keywords:** ultrasound neuromodulation, thermal effects, neuroimaging, calcium imaging, mouse brain

## Abstract

**Significance:**

An array of techniques for targeted neuromodulation is emerging, with high potential in brain research and therapy. Calcium imaging or other forms of functional fluorescence imaging are central solutions for monitoring cortical neural responses to targeted neuromodulation, but often are confounded by thermal effects that are inter-mixed with neural responses.

**Aim:**

Here, we develop and demonstrate a method for effectively suppressing fluorescent thermal transients from calcium responses.

**Approach:**

We use high precision phased-array 3 MHz focused ultrasound delivery integrated with fiberscope-based widefield fluorescence to monitor cortex-wide calcium changes. Our approach for detecting the neural activation first takes advantage of the high inter-hemispheric correlation of resting state ${\mathrm{Ca}}^{2+}$ dynamics and then removes the ultrasound-induced thermal effect by subtracting its simulated spatio–temporal signature from the processed profile.

**Results:**

The focused 350  μm-sized ultrasound stimulus triggered rapid localized activation events dominated by transient thermal responses produced by ultrasound. By employing bioheat equation to model the ultrasound heat deposition, we can recover putative neural responses to ultrasound.

**Conclusions:**

The developed method for canceling transient thermal fluorescence quenching could also find applications with optical stimulation techniques to monitor thermal effects and disentangle them from neural responses. This approach may help deepen our understanding of the mechanisms and macroscopic effects of ultrasound neuromodulation, further paving the way for tailoring the stimulation regimes toward specific applications.

## 1 Introduction

Precise noninvasive neuromodulation can potentially help better understanding the inner workings of the brain and tackle the rise of neurodegenerative diseases in an aging population. Deep brain electrical stimulation, which employs surgical implantation of electrodes, has become a treatment option for several neurological disorders including Parkinson’s disease and epilepsy.^1^^,^^2^ Noninvasive brain stimulation techniques based on electric or magnetic fields are incapable of targeting deeper structures without affecting more superficially located tissues.^3^ In contrast, sound waves can be focused into tiny tissue volumes with minimal collateral effects.^4^ Focused ultrasound (FUS) thus has the potential to noninvasively target nearly any brain area, both in animal models and humans.^5^^–^^8^ Unlike the mechanisms of electric and magnetic field interaction with the neurons, the effects of ultrasonic fields at the cellular, network, and whole brain level have not been fully understood,^9^^–^^12^ chiefly due to the lack of efficient methods for noninvasive real-time observation of ultrasound neuromodulation (USNM) effects.

Understanding how the brain reacts to mechanical stimuli requires a new set of tools as ultrasound (US) produces multiple physical effects, including radiation force, heating, and cavitation.^13^^,^^14^ Despite the repeated evidence of neuronal activation upon US stimuli in isolated neurons and cell cultures,^10^^,^^12^^,^^15^^,^^16^ observing such responses *in vivo* remains challenging, partially due to the lack of consistency in the stimulation parameters used across different studies.^17^ Using mouse models expressing fluorescent calcium sensors^18^ with precise US delivery poses a technical challenge of combining precise FUS delivery with real-time optical imaging.^19^^,^^20^ Most USNM experiments in mice have therefore relied on motor evoked responses generated by directly sonicating large focal areas in the cortical and subcortical regions of the murine brain.^21^ Reduction in latency and increased ${\mathrm{Ca}}^{2+}$ response has been observed following electrical stimulation with FUS pre-treatment.^19^ A thermosensitve ion channel, TRPV1, was sensitized to respond to FUS-induced heating,^22^ yet no direct calcium signal was reported without genetic manipulations.^22^

Recently, we integrated high-resolution FUS delivery and simultaneous widefield fluorescence imaging to achieve and characterize highly precise FUS targeting in a living mouse brain.^23^ However, our initial observations were dominated by the thermal dynamics of fluoro-thermal tags (FTT) or propagating spreading depolarizations, and no localized neural activity has been isolated. Here, we further advance the ability of cortex-wide fluorescence imaging to observe responses to precisely steered localized FUS stimuli by characterizing the activation dynamics and developing a method to separate thermal fluorescence quenching and actual neural responses. While this is, to our knowledge, the first systematic attempt to compensate for thermal events in fluorescence neuroimaging—major thermal events are pervasive in modern neurotechnology, and thus associated confounds are likely present in a myriad of related studies using one- or two-photon imaging.^9^^,^^24^

## 2 Methods

### 2.1 Experimental Setup and Procedures

**Fig. 1:**
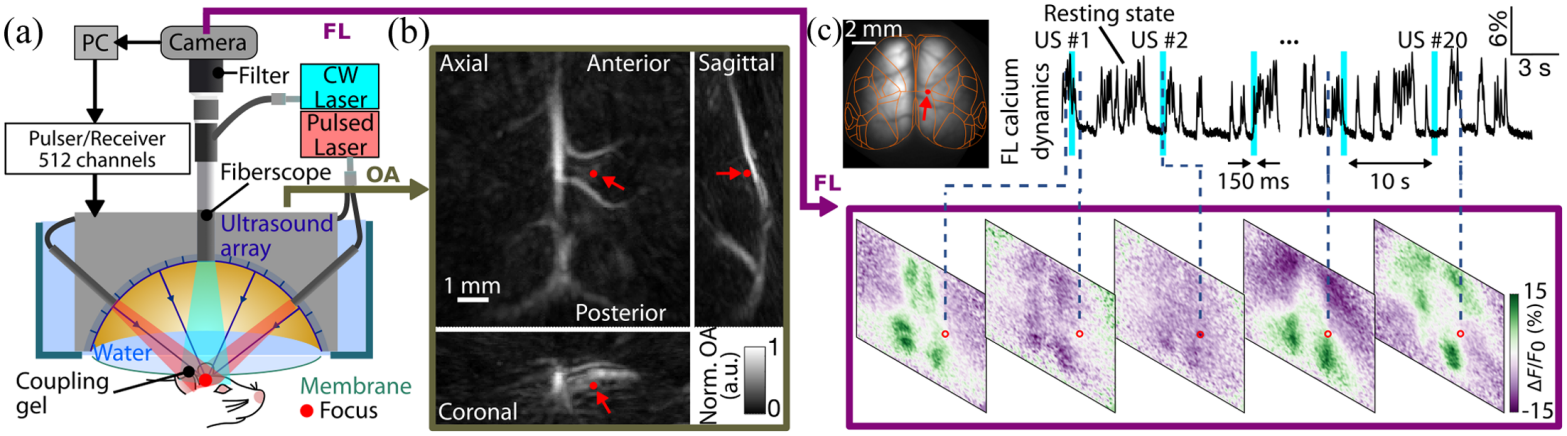
Overview of the FLUS experimental setup and data. (a) Schematic of the multimodal FLUS system. (b) The experimental protocol uses optoacoustic volumetric imaging to precisely navigate the US stimulation to the desired target (red arrow). (c) Fluorescence data are continuously recorded over 20 stimulation cycles (the simultaneous FUS emissions are marked with cyan bars). CW, continuous wave; OA, optoacoustic; US, ultrasound; FL, fluorescent. $\Delta F/F_{0}$ corresponds to relative fluorescence intensity changes to the baseline.

The fluorescence-guided focused ultrasound (FLUS) system has been designed to achieve simultaneous fluorescence imaging and precise noninvasive FUS stimulation of the murine brain. A wide-angle spherical US array (Imasonic, France) consisting of 512 transducer elements having a wide (3 to 9 MHz) effective bandwidth was employed for delivering FUS into the target location [Fig. 1(a)]. The array is capable of generating small focal spots (measuring down to 350  μm) through the mouse skull at any depth and location in the brain.^25^ At the same time, the exact location of the focus can accurately be tracked in three dimensions by means of real-time volumetric optoacoustic tomography (VOT) feedback performed with the same spherical array. For this, excitation of optoacoustic responses is performed with a pulsed laser beam (800 nm wavelength) guided by means of optical fiber bundles to the tissue surface. Absorption of a single 10 ns pulse duration laser pulse by tissue chromophores, such as hemoglobin, triggers the generation of tiny US vibrations, which are detected by the spherical array. The VOT images are then rendered at a real-time frame rate of 25 Hz established by the pulse repetition rate of the laser.^26^^,^^27^ The imaging feedback and US emission are automatically co-registered by the time-reversal principle since both are employing the same transducer array. As a result, the VOT images can be used to precisely navigate the location of the US stimulation target. During the experiments, the array was immersed in deionized water at room temperature and coupled to the sample using a thin (<20  μm thick) polyvinyl chloride film that is transparent to both light and US. Fluorescence imaging was performed simultaneously with the FUS emission through an 8-mm-diameter centrally located hole in the spherical array by means of a flexible fiberscope, attaining an effective 12-mm-diameter circular FOV at 44-μm lateral resolution. A continuous wave laser at 488 nm is used for exciting the GCamp6f calcium-sensitive proteins expressed in the mouse brain.

### 2.2 *In Vivo* Experiments

Seven GCaMP6f mice [C57BL/6J-Tg(Thy1-GCaMP6f) GP5.17Dkim/J, the Jackson Laboratory] were used for this study (three female and four male) aged between 5 and 6.5 weeks. The animals were housed in individually ventilated cages inside a temperature-controlled room, under a 12-h dark/12-h light cycle. Pelleted food (3437PXL15 and CARGILL) and water were provided ad libitum. All experiments were performed in accordance with the Swiss Federal Act on Animal Protection and approved by the Cantonal Veterinary Office Zurich. The mouse head was secured using a custom stereotactic frame (Narishige International Limited, London, United Kingdom) fixed by a holder to minimize motion artifacts for acquiring *in vivo* images during FUS stimulation. Blood oxygen saturation, heart rate, and mouse body temperature were continuously monitored. The core body temperature was maintained at ∼36°C using a homeothermic temperature controller coupled to a heating pad, both of which were controlled by PhysioSuite (Kent Scientific, Torrington, Connecticut).

To ensure optimal US coupling, the hair on the mouse head was removed. We injected buprenorphine (0.1  mg/kg) subcutaneously and removed the scalp after 30 min. A 40% dilution of phosphate-buffered saline in ultrasound gel (Aquasonic Clear, Parker Laboratories Inc., Fairfield, New Jersey) was deposited on mouse’s scalp and brought into contact with the transparent membrane of the tank filled with degassed water to ensure unobstructed transmission of US into the mouse brain for imaging.

All mice were sonicated under isoflurane anesthesia [3% (v/v) for induction and 1.2% (v/v) for maintenance] through an intact skull with 150-ms duration pulses at 3 MHz delivered in the mouse cortex [Fig. 1(c)]. In the repeated FUS stimulation studies peak pressure range was adjusted from 2.5 to 2.8 MPa and the constant time interval between two sequential stimulations was 10 s to minimize interference among consecutive stimulations. The array’s generated pressure at the focus was measured with a 75  μm calibrated hydrophone through a mouse skull. The US intensity can be approximated from the pressure as $I=p^{2}/(2\rho c)$, where p, ρ, and c represent the pressure, density, and speed of sound, respectively.^7^ To test the temporal precision and repeatability of different FUS parameters, we applied 20 repeated stimuli in each experiment. No unusual behavior was observed during the experiments.

### 2.3 *Ex Vivo* Experiment

The brain of one GCaMP 6f mouse was extracted and cut into 1 mm thick slices. The slices are immersed in a temperature-controlled water bath. The temperature was continuously monitored using a thermocouple (IT-23, Physitemp Instruments, Clifton, New Jersey) and recorded to a PC by means of a USB interface (NI 9213, National Instruments, Austin, Texas). Heating and subsequent cooling cycles are averaged together. Fluorescence was recorded using the same setup previously described in Sec. 2.1.

### 2.4 Data Analysis

**Fig. 2:**
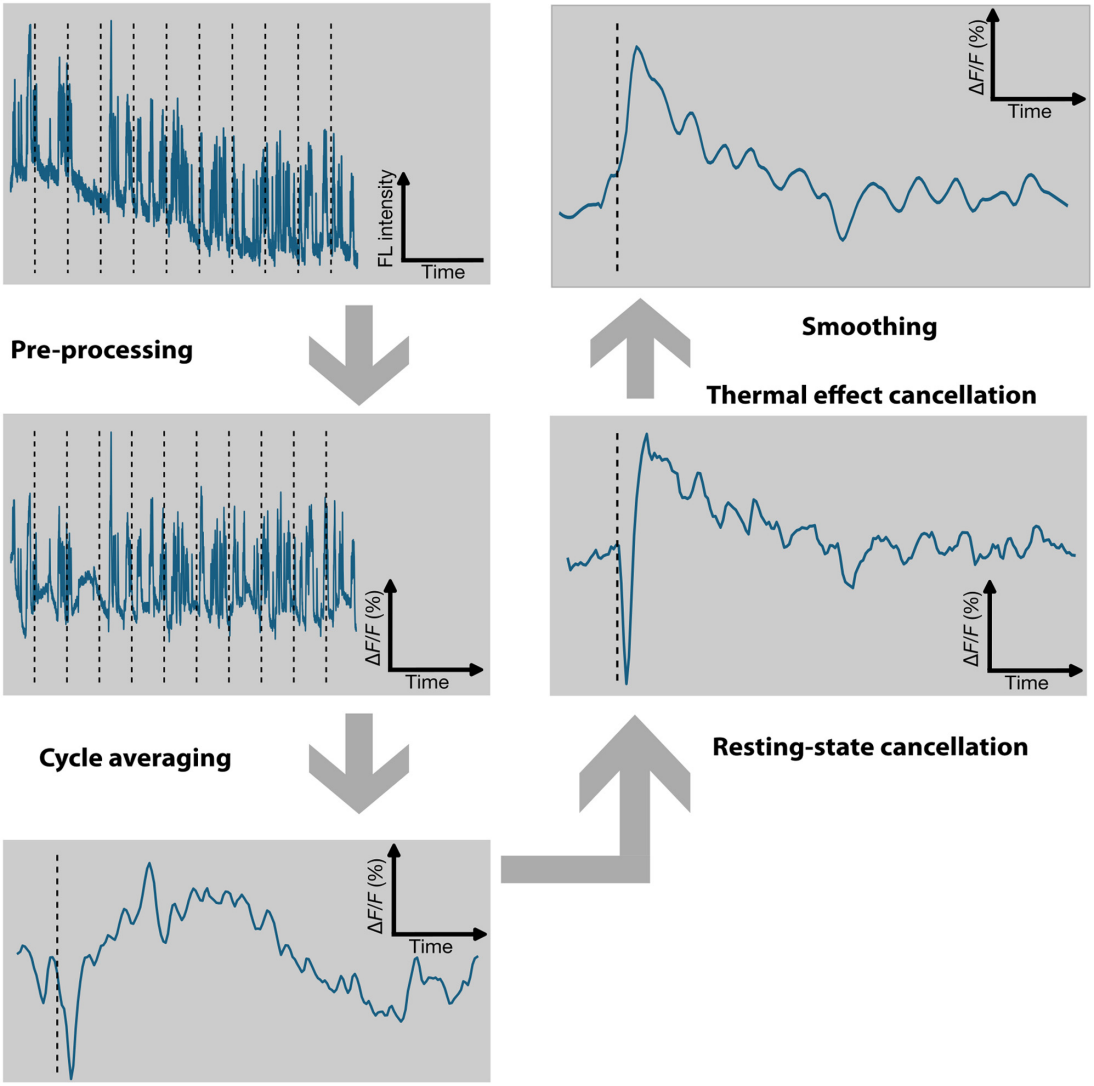
Data pre- and post-processing pipeline. The raw image stack is denoised with a predictive Kalman filter, band-pass-filtered between 0 and 8 Hz and normalized by calculating the relative fluorescence change relative to the moving baseline to remove signal drifts due to laser energy fluctuations or photobleaching. A total of 20 stimulations separated by a period of 10 s are then averaged to cancel noise and remove background from the ${\mathrm{Ca}}^{2+}$ dynamics. An isotropic Gaussian filter is then applied to smooth the image. Signals recorded from the opposite hemisphere to the FUS delivery are subtracted to further increase the SBR. The calculated spatio–temporal signature of the FTT is subsequently subtracted from the processed profile in time and space and temporally smoothed by Savitzky–Golay filter.

The pipeline for analyzing the data is depicted in Fig. 2. The time profiles from fluorescent recordings originate from the same location where the US was emitted and focal FTT-related dip was observed. As a pre-processing step, we denoised the image stack with a predictive Kalman filter in ImageJ with a bias of 0.5 for average sensitivity to momentary fluctuations. The filter is applied on a per-slice basis to the time-lapse sequence of raw fluorescent images (co-registered on the atlas). All other data analyses were conducted using MATLAB (2021b Mathworks, Massachusetts) and custom Python scripts (version 3.10.5). Fluorescence calcium recordings were band-pass-filtered between 0 and 8 Hz and normalized by calculating the relative change to the baseline $\Delta F/F_{0}$ with a moving baseline (0 to 0.05 Hz) to remove signal drifts due to laser energy fluctuations or photobleaching. A total of 20 stimulations separated by a period of 10 s are averaged to cancel noise and remove strong background signal variations due to ${\mathrm{Ca}}^{2+}$ dynamics, which were found to be an order of magnitude larger than the US-induced responses and obscured ${\mathrm{Ca}}^{2+}$ responses from single excitation events. An isotropic Gaussian filter with a kernel size of 1 pixel (∼40  μm) was then applied to smoothen the image. To further increase the signal-to-background ratio (SBR), we took advantage of the high interhemispheric correlation of resting state ${\mathrm{Ca}}^{2+}$ dynamics and subtracted the signals recorded from the opposite hemisphere to the FUS delivery. Activation is localized based on the observed focal FTT dip in the region of interest with high precision in time and space. The calculated spatio–temporal signature of the FTT^23^ was subsequently subtracted from the processed profile in time and space. The processed time profiles were temporally smoothed by Savitzky–Golay filter with a filter window of 11 and polynomial order of 2 to fit the samples. For quantitative analysis, normalized peak amplitude was identified for each profile as the maximum percentage of relative change with respect to the baseline $\Delta F/F_{0}$ during 2 s after the US stimulation onset.

### 2.5 Simulations

FUS simulations are performed assuming linear US wave propagation with the software FieldII.^28^ The simulations were calibrated using hydrophone measurements performed under full water immersion in a water tank.^23^^,^^25^

Simulations of the spatio–temporal heat deposition dynamics induced by FUS are modeled using the bioheat model^29^ as implemented by Soneson.^30^ The input to the model corresponds to the spatial US field distribution using the same parameters used in the experiments with the thermal constants adapted from the literature.^23^^,^^31^

## 3 Results

**Fig. 3:**
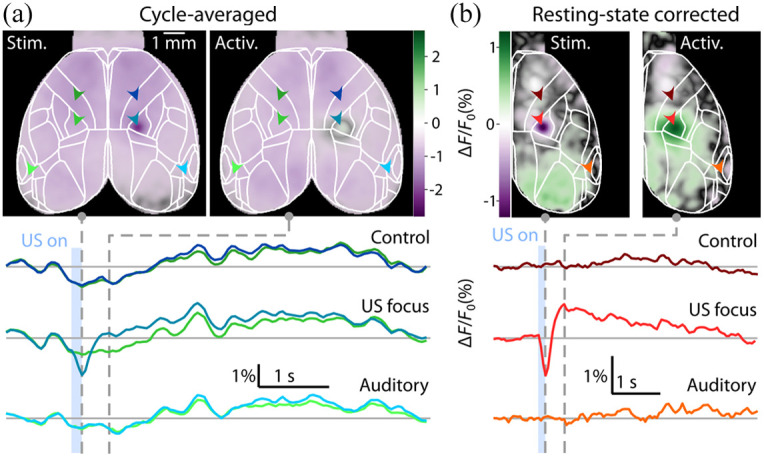
Snapshots of the US-induced fluorescence changes showing the immediate FTT responses and 500 ms thereafter. (a) The data were averaged over 20 stimulation cycles. Color arrows indicate the points where the time traces (below) were extracted. (b) IH subtraction cancels out highly correlated resting state, hence revealing the FTT followed by localized activation in the stimulated area.

In absence of USNM, the mouse brain under isoflurane anesthesia (1.2%) presents spontaneous resting-state calcium dynamics as the background signal [Fig. 1(c)]. Once the FUS pulse is applied, the FTT response occurs almost simultaneously, as indicated by the purple spot [Fig. 3(a), see Fig. S1 in the Supplementary Material for the raw response]. The FTT reflects the position and size of the US focus, and it also precedes the subsequent neural activation event that spreads over a wider area [light green spot in Fig. 3(a)]. However, the resting-state activity of the anesthetized mice creates a strong background visible in the images [light purple, Fig. 3(a)] and fluorescence time traces [Fig. 3(a) below]. Given the high inter-hemisphere (IH) correlation of the resting-state signals, one can subtract the left hemisphere to cancel the resting state and obtain a much clearer view of both the FTT and the subsequent localized activation [Fig. 3(b)]. The red-colored time trace of the raw fluorescence recording at the focal point depicts both events, with a rapidly rising activation following the FTT. No relevant activity was observed near the auditory cortex.

**Fig. 4:**
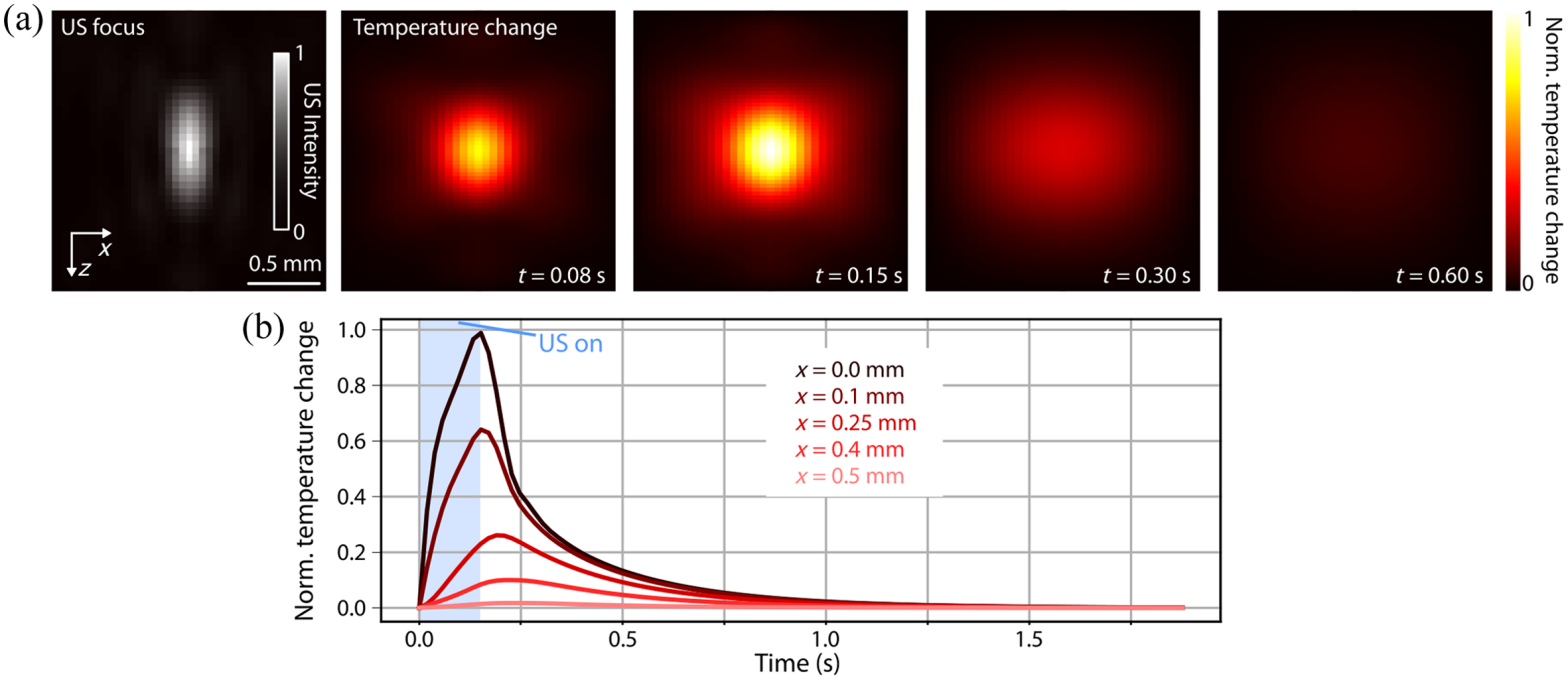
Model-based simulation of thermal effects. (a) US thermal deposition and diffusion simulated using the bioheat model for a continuous 0.15 s duration US pulse. The image on the left shows the simulated US focus on the axial plane, followed by a temporal sequence of the corresponding temperature change. (b) Time evolution of the temperature change extracted at different distances from the US focus (see labels). A blue rectangle marks the sonication time.

To test our hypothesis on the thermal origin of the FTT, we use a bioheat model^23^ to correct for the thermal transients (Fig. 4). The FUS delivery can increase the temperature at the focus and its immediate vicinity [Fig. 4(a)]. The simulated US intensity at the focus and the pulse duration (150 ms) serve as input to the bioheat model to predict the spatio–temporal evolution of the temperature changes. The tightly focused heat source rapidly increases its temperature for the 80 to 150 ms time window, followed by heat diffusion at 300 to 600 ms. A more detailed analysis of the temporal signal evolution at different points surrounding the US focus [Fig. 4(b)] confirms the fast rise and slower decay of the FUS-induced temperature changes.

**Fig. 5:**
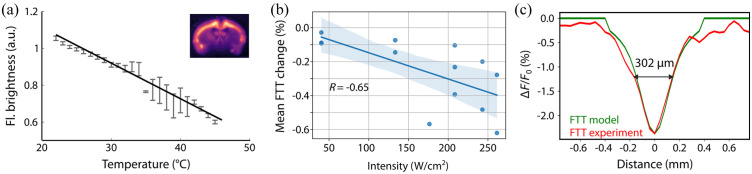
Validation of thermal model versus FTT signal. (a) Change in fluorescence brightness as a function of temperature measured from GCaMP 6f mouse brain slices in a saline solution bath. Experimental data points include error bars corresponding to the standard error of the mean, while the solid line shows an affine fit. (b) The mean of the FTT dip ($\Delta F_{\mathrm{US}}$) during the sonication as a function of US intensity (n=7). Pearson correlation coefficient is indicated as R in the plot. Shaded region corresponds to the 95% confidence interval. (c) Spatial FTT dip in simulation versus experiment.

The temperature dependence of the fluorescence brightness [Fig. 5(a)] has been further validated using *ex-vivo* brain slices of GCaMP-6f-expressing mouse. As expected, an increase in temperature results in quenching of the fluorescence.^23^ The dependence of the mean FTT decay on the FUS intensity [Fig. 5(b)] reveals a negative correlation (Pearson correlation R=−0.65), confirming the US–thermal–fluorescence quenching process. Looking at FTT’s spatial footprint, the model is in reasonable agreement with the experimental data acquired *in-vivo* through the mouse skull.

**Fig. 6:**
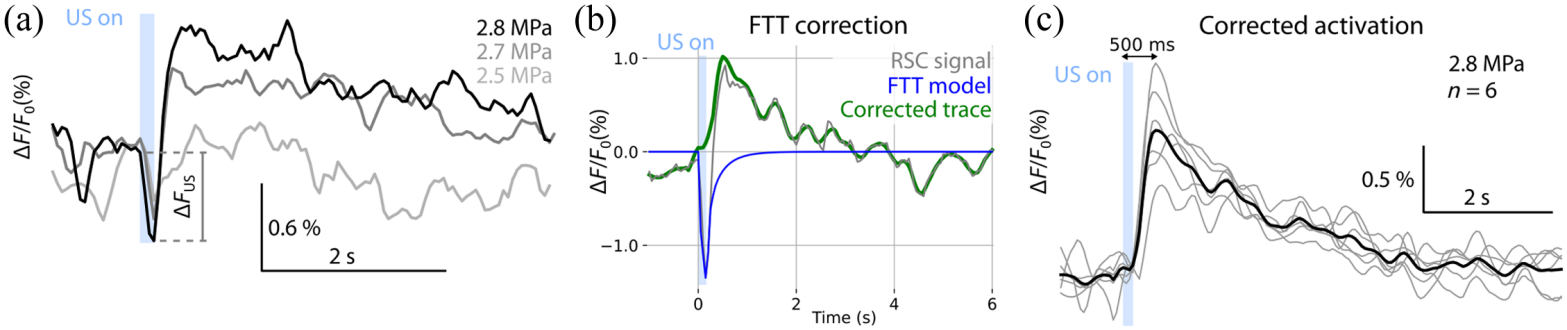
Correction for thermal responses reveals the underlying neural responses to US stimulation. (a) Interhemispheric traces in the stimulated region for different pressures. (b) The IH signal is corrected using thermal model. (c) The corrected US-mediated activation in n=6 animals. Gray curves depict traces from different mice, black curve corresponds to the mean response.

Changing the pressure after IH subtraction generates a pattern of two opposing phenomena [Fig. 6(a)]. On the one hand, we observe the deepening of the FTT followed by a stronger calcium response with the increase in pressure. The simulated thermal transient is subsequently subtracted from the measured ${\mathrm{Ca}}^{2+}$ signal and smoothed to clearly reveal the underlying FUS-evoked activation [Fig. 6(b)]. The activation was robust and consistent among n=6 mice with a peak latency of ∼500  ms [Fig. 6(c)], as measured from the onset of the stimulation to the activation peak. These results are generally in agreement with previous GCaMP6f-based measurements of FUS stimuli *in vitro*^10^^,^^12^^,^^32^^,^^33^ in terms of ${\mathrm{Ca}}^{2+}$ activation rise time and signal shape. We next examined whether the observed responses were stationary across the experiment by comparing the mean responses for the first and last 10 stimulations. Our results (Fig. S2, Table S1 in the Supplementary Material) validate that responses did not fatigue during the experiments.

## 4 Discussion

Our results show that the correction of rapid thermal confounds is a potentially crucial and feasible step toward direct evidence of neuronal network activation upon precise FUS stimulation *in vivo*. The significance and benefit of this processing solution is high: precise stimulations in both lateral and axial dimensions together with FTT-guided FUS delivery overcomes the limitations of previous studies.^34^ FTT-guided FUS delivery rules out such confounds as it can monitor precisely the US delivery location.

Our study found that relatively high pressure levels are needed to activate the mouse brain at the 3 MHz frequency used in our experiments. The detection sensitivity and resolution limits of the imaging system may have limited our ability to measure weak activations at lower pressures. Nonetheless, in contrast to other high resolution ${\mathrm{Ca}}^{2+}$ imaging approaches over restricted (sub-millimeter) field of view, cortex-wide fluorescence allows minimizing the resting state background thus obtaining cleaner activation traces. Previous studies reported lower activation thresholds with significantly longer stimulations, e.g., for neurons over-expressing the TRPV1 ion channel where stimulation durations in the 7 s range were used.^22^ The relatively high neural activation thresholds can arguably also be attributed to the high temperature increase in the targeted spot, which in turn may have caused inhibitory effect thus partially canceling out excitatory effects.^33^^,^^35^^–^^40^ This balance of excitatory and inhibitory events is probably omni-present in this type of experiments and remains to be carefully explored using the tools introduced here. The 3 MHz excitation frequency used in our study produces obvious transient thermal effects and higher radiation forces as compared to lower frequencies. Conversely, the probability of inducing cavitation is also lower^41^ at maximum mechanical index (MI) of 1.6 used in our study, i.e., below the FDA-required safety limit of 1.9 for diagnostic US imaging.

Photobleaching and laser heating due to the continuous wave laser excitation (see Sec. 2.1) are present in the ${\mathrm{Ca}}^{2+}$ signals but occur on much longer time scales than the transient ultrasonic heating. Therefore, baseline correction and cycle averaging should be used to remove slow photobleaching and thermal effects from the ${\mathrm{Ca}}^{2+}$ signals.

Future work should aim at characterizing specific regimes for optimal FUS stimulation under specific experimental conditions and application-related requirements. Our flexible image-guided platform enables systematic testing over a wide parameter space in various brain regions. Due to the bulky arrayed US transducer setup, animal studies are mostly limited to stimulation under anesthesia or heavy sedation, which typically suppress the neural response to stimulation,^11^ or otherwise to head-restrained, awake animals. Furthermore, deep learning methods can be developed and integrated into the analysis pipeline for spatiotemporal enhancement and denoising of calcium imaging responses.^42^

## 5 Conclusion

This study introduced a non-invasive US stimulation technique with precise volumetric optoacoustic navigation and simultaneous fluorescence calcium recordings of the cortical responses. The method can target deep murine brain regions with high spatiotemporal resolution thus holding promise to advance the study of the nervous system and uncover new ways to treat neurological disorders. In addition, the careful handling of thermal confounds is crucial to the understanding of the stimulation processes and clearly differentiate between thermal and neural responses. We expect our method could also find application in other neurostimulation modalities that cause thermal transients and rely on fluorescence as readout of neural responses. Future studies will evaluate various underlying phenomena over a wide range of parameters.

## Data Availability

The data that support the findings of this study are available from the corresponding author upon reasonable request.
