# Supplementary material for: Model-based correction of rapid thermal confounds in fluorescence neuroimaging of targeted perturbation
Source: Neurophotonics. 2024 Feb 16;11(1):014413. doi: 10.1117/1.NPh.11.1.014413 (PMC10871046; doi:10.1117/1.NPh.11.1.014413)
Supplement: Supplementary file 1 [file NPh_011_014413_SD001.pdf]

Supplementary material

Model-based correction of rapid thermal confounds in fluorescence neuroimaging of targeted perturbation

Neda Davoudi[a,b,d,*], Hector Estrada[a,b,*], Ali Özbek[a,b], Shy Shoham[c,+], Daniel Razansky[a, b, d+]

[a] Institute for Biomedical Engineering and Institute of Pharmacology and Toxicology, Faculty of Medicine, University of Zurich, CH-8057 Zurich, Switzerland

[b] Institute for Biomedical Engineering, Department of Information Technology and Electrical Engineering, ETH Zurich, CH-8093 Zurich, Switzerland

[c] Department of Ophthalmology and Tech4Health and Neuroscience Institutes, NYU Langone Health, New York, NY 10016, USA

[d] Currently at ETH AI Center, CH-8092 Zurich, Switzerland

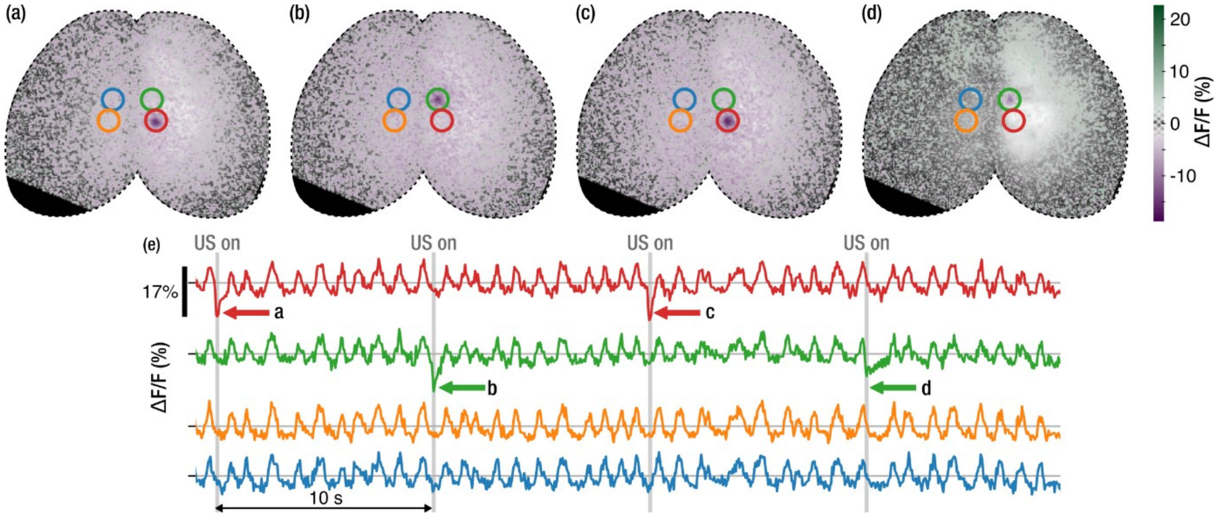

Supplementary Fig. 1 (a) – (d) Snapshots of the widefield fluorescence recording before data processing. Each panel shows the end of the ultrasound sonication showing the fluorothermal tag (dark purple) which appears at the position of the ultrasound delivery. Using the beam-steering capabilities of the spherical ultrasound array, the position of the sonication can be alternated. **(e)** Time traces obtained at the points in the colored circles in (a) – (d). Labels indicate the time of the snapshots.

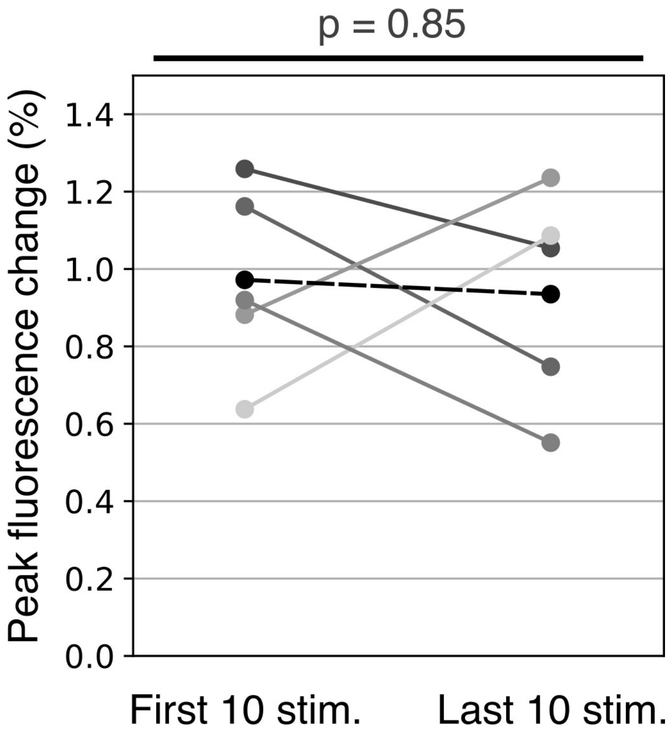

Supplementary Fig. 2: Stimulation fatigue analysis separating the 20 stimulations in two groups, first and last 10 for n = 5 mice. The mean value (black) is connected by dashed lines. A paired t-test confirms there is no statistical difference between both groups using the peak fluorescence change (see Supplementary Table 1).

Supplementary Table 1: Data corresponding to Supplementary Fig. 2.

Peak fluorescence change (%)

Mice Nº	First 10 stimulations	Last 10 stimulations
1	1.26	1.05
2	1.16	0.75
3	0.88	1.24
4	0.64	1.09
5	0.92	0.55
t statistic	0.20	
p-value	0.85	
